# Diversity of Microbial Communities in Production and Injection Waters of Algerian Oilfields Revealed by 16S rRNA Gene Amplicon 454 Pyrosequencing

**DOI:** 10.1371/journal.pone.0066588

**Published:** 2013-06-21

**Authors:** Nesrine Lenchi, Özgül İnceoğlu, Salima Kebbouche-Gana, Mohamed Lamine Gana, Marc Llirós, Pierre Servais, Tamara García-Armisen

**Affiliations:** 1 Department of Biology, Laboratory of Conservation and Valorisation of Biological Ressources, University M’Hamed Bougara of Boumerdes, Boumerdes, Algeria; 2 Ecology of Aquatic Systems L, Université Libre de Bruxelles, Brussels, Belgium; 3 Center of Research and Development, Biocorrosion Laboratory (Sonatrach), Boumerdes, Algeria; 4 Department of Genetics and microbiology, Biosciences Faculty, Universitat Autònoma de Barcelona, Bellaterra, Spain; Missouri University of Science and Technology, United States of America

## Abstract

The microorganisms inhabiting many petroleum reservoirs are multi-extremophiles capable of surviving in environments with high temperature, pressure and salinity. Their activity influences oil quality and they are an important reservoir of enzymes of industrial interest. To study these microbial assemblages and to assess any modifications that may be caused by industrial practices, the bacterial and archaeal communities in waters from four Algerian oilfields were described and compared. Three different types of samples were analyzed: production waters from flooded wells, production waters from non-flooded wells and injection waters used for flooding (water*-*bearing formations). Microbial communities of production and injection waters appeared to be significantly different. From a quantitative point of view, injection waters harbored roughly ten times more microbial cells than production waters. *Bacteria* dominated in injection waters, while *Archaea* dominated in production waters. Statistical analysis based on the relative abundance and bacterial community composition (BCC) revealed significant differences between production and injection waters at both OTUs_0.03_ and phylum level. However, no significant difference was found between production waters from flooded and non-flooded wells, suggesting that most of the microorganisms introduced by the injection waters were unable to survive in the production waters. Furthermore, a Venn diagram generated to compare the BCC of production and injection waters of one flooded well revealed only 4% of shared bacterial OTUs. Phylogenetic analysis of bacterial sequences indicated that *Alpha-*, *Beta-* and *Gammaproteobacteria* were the main classes in most of the water samples. Archaeal sequences were only obtained from production wells and each well had a unique archaeal community composition, mainly belonging to *Methanobacteria*, *Methanomicrobia*, *Thermoprotei* and *Halobacteria* classes. Many of the bacterial genera retrieved had already been reported as degraders of complex organic molecules and pollutants. Nevertheless, a large number of unclassified bacterial and archaeal sequences were found in the analyzed samples, indicating that subsurface waters in oilfields could harbor new and still-non-described microbial species.

## Introduction

Petroleum reservoirs are complex ecosystems located in deep geological formations: they are anoxic and often characterized by high temperature, pressure, and salinity [1]. Due to these extreme conditions, which are challenging for most life forms, petroleum reservoirs were formerly considered sterile. In 1926, for the first time, Bastin [2] demonstrated the presence of sulfate-reducing microorganisms in oilfields. Today, with the currently available molecular tools, we know that these anaerobic ecosystems harbor a wide variety of microorganisms that have successfully adapted to the prevailing extreme physicochemical conditions [1].

After 100 years of exploitation, these ecosystems have also been subjected to anthropic modifications. Nevertheless, little is known about the impact of industrial practices on the petroleum microbial community. Among various processes developed to enhance oil recovery, water, gas or chemical injection are the most widely used. Their purpose is to increase the pressure in the well in order to facilitate oil rising (for a review see [3]). Previous studies have shown that the injected waters generally taken from the surface present a large microbial community that is different from that found in autochthonous well water; it was therefore expected that the water flooding process would modify the microbial community in the reservoir [4], [5].

Studying these multi-extremophiles is not only fascinating, but also important from an economic point of view as they could severely affect oil quality and reservoir permeability. Some of the potential impacts of the microbial activity are the increase of oil density and viscosity, the increase of sulphur and metal content, reservoir souring, acidification [6] and microbiologically influenced corrosion (MIC) [7]. In particular, sulphides generated by sulphate-reducing *Bacteria* (SRB) could be responsible for up to 80% of all corrosion damage to oil field operating machinery [7] causing severe economic losses. On the other hand, the positive effects of microbial communities can be exploited by studying the activity and metabolism of oil reservoir microbial communities. Microorganisms are being used to improve the production by enhanced oil recovery (EOR) and prevent reservoir souring. In addition, many microorganisms isolated from oil reservoirs are able to produce bioproducts such as biosurfactants [8], biopolymers [9], solvents, acids and gases [10].

A great variety of microorganisms have been described from a number of geographically distant oil reservoirs; including sulphate reducers [11], sulphidogens [12], fermentative *Bacteria* [13], methanogens [14], acetogens [15] and manganese and iron reducers [16], [17]. These microorganisms have a wide range of growing temperatures, from mesophilic (15–40°C) to thermophilic (45–80°C) [17], [18], [19].

Culture-based methods have been extensively applied in the field of petroleum microbiology [13], [20]. However, due to their multi-extremophilic lifestyles only a very small fraction of the microbial diversity can be accessed using culture-based methods. In more recent studies, culture-independent approaches such as 16S rRNA gene cloning and sequencing, denaturing gradient gel electrophoresis (DGGE) and terminal restriction fragment length polymorphism (T-RFLP) have been successfully used providing insights into the uncultivated microbial communities in subsurface petroleum reservoirs [1], [4], [5], [21], [22], [23]. Nevertheless, the traditional molecular methods are often limited to the analysis of a relatively small number of clones and thus only a small fraction of the microbial diversity has been unraveled by the previous studies. During the last 5 years, new technologies have been developed that provide better access to microbial diversity. Tag-encoded FLX amplicon pyrosequencing (TEFAP), developed by Roche 454 Life Science (Branford, CT, USA), is a technique making possible to obtain high numbers of DNA reads through a massively parallel sequencing-by-synthesis approach [24]. This technology is now widely used to analyze the microbial community in various types of environmental samples, such as soil [25] and marine water [26]. However, to the best of our knowledge, until now only two studies have been published on the microbial diversity of Canadian oilfield using TEFAP [27], [28].

The objective of the present study was to describe and compare the phylogenetic diversity of the microbial communities in various production and injection wells in Algerian oilfields. Eight samples (five production wells and three injection wells) from four different sites (Tin Fuin Tabankort, Stah, Bir Rabie Nord and Ouhanet) were studied. These wells differ from one another and from other wells already reported in the literature by their location, depth, temperature, salinity, stratigraphic distribution and whether or not water flooding is present. To the best our knowledge, all the available studies on microbial populations in oilfields have focused on high- and low-temperature petroleum reservoirs; and none of them present an array of samples covering a range of different salinities and pH.

The samples collected were first analyzed with the Catalyzed Reporter Deposition Fluorescence In Situ Hybridization (CARD-FISH) technique using probes targeting both the *Archaea* and *Bacteria* domains to determine the relative abundance of each group. Then a 16S rRNA gene amplicon 454 pyrosequencing approach was used to analyze the structure and diversity of the microbial communities in greater detail. Correlations between microbial community compositions and water physicochemical characteristics were also investigated.

## Materials and Methods

### Study Sites and Sampling Strategy

Sonatrach (Société Nationale Algérienne pour la Recherche, la Production, le Transport, la Transformation et la Commercialisation des Hydrocarbures) allowed us to sample the oilfields described below. Eight water samples were collected in May 2011 from four different oilfields located in the southern Algerian Sahara: Tin Fuin Tabankort (T), Stah (S), Bir Rabie Nord (BD) and Ouhanet (OH). At each sampling site, different wells were sampled: PNFT1 (PNF for production water from a non-flooded well), PNFT2 and IT3 (I for injection water) from the T site, PFS1 (PF for production water from a flooded well) and IS2 from the S site, IBD from the BD site (no production water sample was available at this site) and PFOH1 and PFOH2 from the OH site (no injection water sample was available at this site).

Twenty liters of water were collected in sterile jerry cans directly from the wellhead of each well sampled, completely filled and sealed directly with screw caps to avoid contamination and oxygen intrusion. The samples were immediately transported at ambient temperature to the laboratory and stored at 4°C until analysis. All samples were treated within 24 h after collection. Temperature, pH and salinity were measured *in situ* using a multi-parameter probe (Hanna Instruments, Smithfield, RI, USA). The analytical methods used in this study to measure ions concentration (Ca^2+^, Mg^2+^, Fe^2+^, Na^+^, K^+^, Ba^2+^, Cl^−^, HCO_3_^−^, SO_4_^2−^) were based on 4500-S-2 F standard methods, as described before [29].

### Catalyzed Reporter Deposition-Fluorescence in situ Hybridization (CARD-FISH)

For cell enumeration purposes, water samples (100 mL) were first fixed with paraformaldehyde (PFA) (2% final conc., adjusted to pH 7.4). Within 2 h after fixation, samples were filtered onto white polycarbonate filters (Millipore, USA, Type GTTP; pore size, 0.2 µm; diameter, 47 mm), washed twice with phosphate-buffered saline (PBS) buffer (pH 7.6), dried, and stored at −20°C until further processing. Total cell numbers were determined by epifluorescence counting after staining with 4′, 6-diamidino-2-phenylindole (DAPI) as previously described [30]. *Bacteria* and *Archaea* were enumerated by CARD-FISH using the specific probes EUB338 and ARCH915 labeled with Alexa-Fluor_488_ (Table 1), using the protocol described by Teira *et al.* [33] with slight modifications to improve cell wall permeabilization. Cell losses during permeabilization and filter processing were minimized by dipping the filters in low-gelling-point agarose (0.1% [w/vol], in Milli-Q water) and drying them upside down in a glass petri dish at 37°C. The filters were subsequently dehydrated in 95% ethanol (vol/vol) and allowed to air dry. To inhibit potentially present intracellular peroxidases, filters were incubated with 0.01 M HCl at room temperature (RT) for 20 min, washed with PBS 1× buffer and Milli-Q water and dried. For cell wall permeabilization, filters were incubated with lysozyme solution (10 mg/mL in 0.05 M EDTA, and 0.1 M Tris-HCl at pH 8.0; Sigma) for 30 min at 37°C and, afterwards gently rinsed with Milli-Q water and absolute ethanol. Filters were then incubated with proteinase K (0.25 U/mg, concentration, 0.25 mg/mL in 0.05 M EDTA and 0.1 M Tris-HCl [pH 8.0]; Fermentas, Hanover. MD, US) for 5 min at 50°C and washed with Milli-Q water. Subsequently, the filters were incubated with 4% PFA (w/vol, final concentration) for 5 min at room temperature, washed with Milli-Q water, dehydrated with absolute ethanol, and air dried. Probe hybridization, washing, signal amplification, and filter preparation were performed as described previously [33], except that 55% (vol/vol) formamide was used for both probes. Finally, filter sections were air-dried and examined under an epifluorescence microscope (DM RXA*;* Leica Microsystems, Wetzlar, Germany) equipped with a 50-W Hg bulb and appropriate filter sets for DAPI and Alexa-Fluor_488_. Triplicate filters were processed independently for each sample. At least 20 microscopic fields were randomly selected to count DAPI-stained and probe-hybridized cells.

**Table 1:** Probes and primers used in this study in CARD-FISH and 16rRNA gene amplicon 454 pyrosequencing.

Process	Probe/Primer name	Target	Sequence (5′–3′)	References
CARD-FISH	EUB338	Most *Bacteria*	gCTgCCTCCCgTAggAgT	[31]
	ARC915	*Archaea*	gTgCTCCCCCgCCAATTCCT	[32]
454 Pyrotag sequencing	27F	Bacterial 16S	GAGTTTGATCNTGGCTCAG	[39]
	519R	Bacterial 16S	GWNTTACNGCGGCKGCTG	[40]
	Arch349F	Archaeal 16S	GYGCASCAGKCGMGAAW	[41]
	Arch806R	Archaeal 16S	GGACTACVSGGGTATCTAAT	[41]

### DNA Extraction Method

Microbial biomass was collected from water fraction and concentrated by filtration. An aliquot (from 0.8 L to 5.0 L) of each sample was filtered in triplicate directly through the 0.2-m pore size, 47-mm-diameter polycarbonate filters (Millipore, Billerica, MA, USA). All filters were then placed in 3 mL lysis buffer solution (50 mM Tris-HCl, pH 8.0, 40 mM EDTA, 750 mM sucrose) and stored at −20°C until use. DNA was extracted using two methods in parallel: one based on enzymatic cell lysis (lysozyme and mutanolysin) followed by hot detergent lysis [34], [35] and another one based on the use of cetyltrimethyl ammonium bromide (CTAB) as previously described [36]. Dry DNA pellets were finally rehydrated in 100 L of 10 mM Tris-HCl buffer (pH 7.4) and further purified using the Min Elute reaction cleanup kit (QIAGEN). DNA concentration and purity were then determined using a Nanodrop ND-2000 UV-Vis spectrophotometer (Nanodrop, Wilmington, DE, USA). Purified DNA extracts were stored at −20°C until use. In order to minimize putative bias due to the DNA extraction methods used, the DNA obtained by both methods were pooled together in equal concentrations before being sent for sequencing.

### Bacterial and Archaeal 16S rRNA Gene Tag-encoded FLX-titanium Amplicon Pyrosequencing

Bacterial and archaeal tag-encoded FLX gene amplicon pyrosequencing (bTEFAP and aTEFAP, respectively) analysis were carried out by means of a Roche 454 FLX instrument with titanium reagents. Titanium procedures were performed at the Research and Testing Laboratory (Lubbock, TX, USA) based upon RTL protocols (www.researchandtesting.com) as previously described [37], [38]. DNA samples were diluted to a final concentration of 20 ng/µL prior to bTEFAP and aTEFAP. The PCR primers for FLX amplicon pyrosequencing were chosen to span the variable V1–V3 regions in the 16S rRNA gene: 27F (5′-GAGTTTGATCNTGGCTCAG-3′) and 519R (5′-GWNTTACNGCGGCKGCTG-3′) for *Bacteria* and ARCH 349F *(*5′-GYGCASCAGKCGMGAAW-3′) and ARCH 806R (5′-GGACTACVSGGGTATCTAAT-3′) for *Archaea* (Table 1). These selected primers cover about 78% and 70% of publicly available 16S rDNA sequences for *Bacteria* and *Archaea*, respectively (TestPrime tool at SILVA webpage http://www.arb-silva.de/search/testprime/).

The raw reads of both runs (*Bacteria* and *Archaea*) have been deposited under the sequence read archive (SRA) section of GenBank with the following accession numbers for each sample: SRX265152 (PNFT1), SRX265153 (PNFT2), SRX265483 (IT3), SRX265484 (PFS1), SRX265485 (IS2), SRX265486 (IBD), SRX265487 (PFOH1), SRX265488 (PFOH2).

### 16S rRNA Data Processing

The open-source, platform-independent, community-supported software program, Mothur (Mothur v.1.25.1; http://www.mothur.org) [42] was used to process and analyze the sequence data. To minimize the effects of random sequencing errors, a denoising algorithm included in the pipeline is used and low-quality sequences were removed by eliminating those without an exact match to the forward primer, without a recognizable reverse primer, with a length shorter than 200 nucleotides and those containing any ambiguous base calls. We trimmed the barcodes and primers from the resulting sequences. Chimeric sequences were also removed using Mothur.

We clustered the sequences into operational taxonomic units (OTUs) by setting a 0.03 distance limit (equivalent to 97% similarity). Rarefaction curves based on identified OTU, Shannon diversity index and species richness estimator Chao1 were generated using Mothur for each sample. Sequences were classified using the greengenes database at a 80% confidence threshold with Mothur. After phylogenetic allocation of the sequences down to the phylum, class and genus level, relative abundance of a given phylogenetic group was set as the number of sequences affiliated with that group divided by the total number of sequences per sample. Venn diagrams of unique and OTUs (0.03 cut-off value) were drawn in order to highlight similarities and shared sequences between the different analyzed samples.

### Statistical Analyses

A data matrix was created based on the relative abundance of OTUs (0.03 cut-off) level and subsequently square-transformed, and the Bray-Curtis similarity coefficient was used to quantify resemblances. Multidimensional scaling ordination (MDS) analyses were performed using PRIMER 6 [43] and plotted to assess similarities among samples in two-dimensional space. MDS plots represent relative distances among samples in relation to the rank order of their relative similarities. In the MDS plots, sample points that are close together are more similar in their bacterial composition than those that are far apart. The goodness-of-fit of the plot to the similarity matrix was assessed with a stress formula. Stress values less than 0.2 indicate a good representation of the data in a 2D space [44]. Procrustes analyses were done to determine the degree of concordance between MDS ordinations obtained at different taxonomic resolution levels (phylum and OTU_0.03_) using R software with Vegan package (R development).

The Spearman correlation test was used to analyze the correlations between relative abundance of OTUs (97%) and phylum/genera/environmental parameters. The discrimination of bacterial assemblages based on water type was tested with one-way analysis of similarities (ANOSIM) using PRIMER 6 [43].

## Results and Discussion

### Physicochemical Characterization of the Samples

The geological characteristics of the wells studied and the physicochemical properties of the collected water samples are reported in Table 2. The properties of the different samples differed considerably: production waters (PNFT1, PNFT2, PFOH2, PFOH1 and PFS1,) had higher temperatures (from 87°C to 96°C) than injection waters (IS2, IT3 and IBD; 51°C to 64°C) and pH values ranged from 4.50 to 7.11 for production waters and from 7.08 to 8.33 for injection waters. Total salinity of non-water-flooded oilfields was 250.80 g/L and 189.95 g/L for PNFT1 and PNFT2, respectively; these salinity values were five and seven times higher than the average salt content of seawater. Salinity was much lower in production waters from flooded wells (PFS1, PFOH2 and PFOH1; from 5.84 to 36.30 g/L) and in injection waters (IS2, IT3 and IBD; from 0.58 to 21.18 g/L). Concentrations of Ca^2+^, Mg^2+^, Na^+^, K^+^, Ba^2+^ and Cl^−^ were also higher in production waters from non-water-flooded reservoirs than in production waters from flooded reservoirs and injection waters. In contrast, high concentrations of SO_4_^2−^ ions were measured in injection waters and in production waters from water-flooded wells, while SO_4_^2−^ was undetectable in production waters from non-flooded wells.

**Table 2:** Well characteristics and physicochemical properties of waters from oil areas in southern Algeria.

	PNFT1	PNFT2	PFOH1	PFOH2	PFS1	IS2	IT3	IBD
**Category** *	P	P	P	P	P	I	I	I
**Depth (m)**	2053	2104	2300	2351	2207	803	1205	930
**Geological period**	Ordovician	Ordovician	Devonian	Devonian	Devonian	Albian	Lias	Albian
**Water flooding**	No	No	Yes	Yes	Yes	–	–	–
**T (c°)**	87	89	96	98	93	51	64	55
**pH**	4.60	4.50	6.90	7.11	6.08	8.02	8.33	7.08
**Salinity (g/L)**	250.80	189.95	5.84	7.30	36.30	0.58	6.58	21.18
**Ca^2+^ (mg/L)**	32501	30110	641.28	577.20	3106	41.88	922	1186.40
**Mg^2+^(mg/L)**	2589	2371	262.65	291.80	1824	13.62	398	856.30
**Fe^2+^(mg/L)**	108	133	2066	0.04	28.08	2.35	0	4.69
**Na^+^(mg/L)**	39039	32340	2300	1825	7232	205.10	751	4500
**K^+^(mg/L)**	1779	1650	356.30	161	378.40	20.21	51	110
**Ba^2+^(mg/L)** **	1100	1094	bd	bd	bd	bd	bd	bd
**Cl^-^(mg/L)**	152000	115235	3545.70	4432.10	22000	354.57	3989	12853
**HCO_3_^-^(mg/L)**	129	11.59	18.30	212.90	88.45	151.89	237	119.56
**SO_4_^2-^(mg/L)** **	bd	bd	503.40	459.20	371	224	952	2897

* **P**: production water**, I**: injection water.

** **bd**: below detection limit.

These results highlight the differences between the physicochemical characteristics of waters from the flooded and non-flooded wells sampled. Water characteristics of non-flooded wells, sampled in this study (high temperature, neutral to acidic pH, high salts, chloride-type and alkaline cations such as calcium and barium) were consistent with the previously reported data in others oilfields [20], [45], [46].

Contrary to what was reported for other oil reservoirs studied [4], [5], it is a common practice in Algeria to use injection waters from aquifers instead of surface waters. Gana *et* *al*. [46] reported that injection waters sampled from Algerian oilfields were characterized by mesophilic temperature, low salinity, neutral pH and high concentration of SO_4_^2−^; this is consistent with our data.

The physicochemical characteristics of the three different types of waters analyzed in the present work (injections, productions from water-flooded wells and productions from non-water flooded wells) were clearly different. Production waters from water-flooded wells show intermediate characteristics between the two others.

### Bacterial and Archaeal Abundance Assessed by CARD-FISH

The total cells enumerated by epifluorescence microscopy after DAPI staining varied from 1.01 × 10^4^ to 4.25 × 10^4^ cells/mL in production waters and from 1.06 × 10^5^ to 1.68 × 10^5^ cells/mL in injection waters (Table S1). These abundance levels are within the range of those previously reported for oil reservoirs [47], [48]. Although the autochthonous waters of flooded wells were mixed with waters containing 10 times more cells, no clear differences were observed between the abundance of cells in production waters from flooded and non-flooded wells.

**Figure 1:**
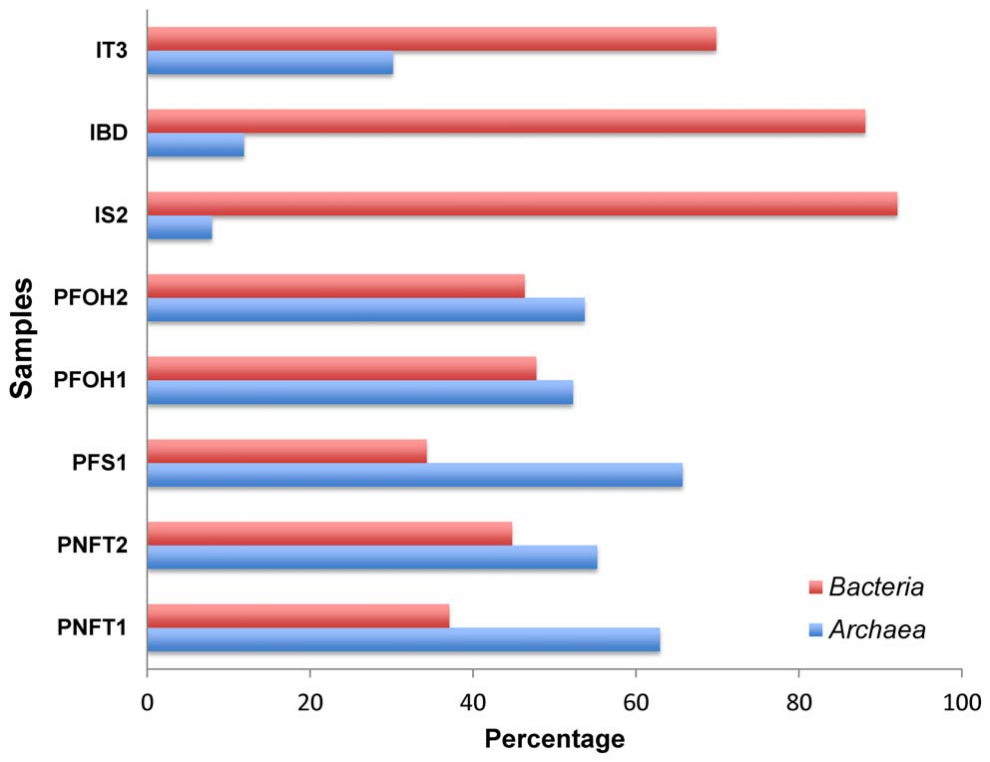
Relative abundance of *Bacteria* and *Archaea* assessed by CARD-FISH. Percentages are calculated considering the sum of the cells hybridized with bacterial and archaeal probes as the total cells numbers.

The abundance of *Bacteria* and *Archaea* was determined using specific CARD-FISH probes (Table S1) in order to estimate the relative contribution of both domains to the microbial community in each sample. The addition of the counts of bacterial and archaeal cells represent only a small percentage of the total DAPI counts. Lower cells counts using the CARD-FISH protocols could be explained by unspecific DAPI staining of dead or non-nucleotid containing cells (ghosts) [49] or cell permeability problem to CARD-FISH probes [50]. For a clear view of the relative contribution of *Bacteria* and *Archaea* to the microbial community, Figure 1 presents the relative abundance of each group considering the sum of the cells hybridized with bacterial and archaeal probes as the total cells numbers. Relative abundance of *Bacteria* in injection waters ranged from 70% to 92%, whereas they never reached values higher than 48% in production waters. *Archaea* relative abundance was thus higher in samples of production waters (ranging from 52% to 66%) than in the injection waters (ranging from 8% to 30%).

As for the total microbial cell counts, the relative abundance of *Bacteria* and *Archaea* in samples from the flooded wells did not differ from those measured in non-flooded well samples. The higher relative abundance of *Archaea* in production water than in injection water is probably related to the extreme physicochemical conditions in this type of wells. It is well known that extremophilic microorganisms that are adapted to harsh environmental conditions such as high temperature, extreme acidic or alkaline pH, high salt, or a combination of these (such as in petroleum reservoirs) mainly belong to *Archaea* [51] with the exception of a few extremophilic *Bacteria*.

It is no worth to mention that, despite the differences in the physicochemical characteristics of waters from flooded and non-flooded wells, no marked differences were observed in either the total microbial counts or in the relative proportion of *Bacteria* and *Archaea*. These results may suggest that environmental factors such as temperature and pressure, which are directly related to depth, might be the main selective factor for the microbial community.

### 16rRNA Gene Amplicon 454 Pyrosequencing Data and Statistical Analyses

The bacterial community composition (BCC) was analyzed by V1–V3 16S rRNA gene region TEFAP analysis. A total of 8064 bacterial sequences were obtained from eight samples. The read numbers were uneven, ranging from 239 to 1575 per sample (Table S2A). As species richness increases with the number of sequences in a given sample, we subsampled all the samples using Mothur randomly to the same size based on the sample with the smallest sequences number. Although observed species and CHAO1 estimations have changed numerically, our main conclusion was similar to the one obtained with the original dataset. Therefore we presented here our original data. Rarefaction analysis based on OTUs at 0.03 cutoff level (Fig. S1A) indicated that most of the bacterial samples might require a stronger sequencing effort to avoid microbial diversity underestimation. Furthermore, considering all the samples, a total of 1499 OTUs (defined using a 0.03 cutoff value) were found. The number of observed OTUs per sample ranged from 11 to 331 in production waters and from 144 to 309 in injection waters (Table S2A). The CHAO1 estimator predicted richness values in the range of 14 to 526 for production waters and 472 to 757 for injection waters. Moreover, obtained OTUs had a Shannon diversity index value of 0.94 to 5.16 for production waters and 3.82 to 4.28 for injection waters. These results indicate that except for PNFT1 sample, the bacterial communities of injection waters were more diverse and richer than those of production waters. Greater richness in the injection waters compared to production waters has been reported previously in Chinese oilfields [5] and oil samples with high-water content have been reported as the most diverse [52]. However, the relative richness of the injection versus production waters depends on the origin of the injection waters but, in general, a more extreme environment is expected to maintain lower species diversity [53] by selecting a small group of highly specialized organisms able to survive and grow in such challenging environments [54]. In this study, the major differences in environmental conditions, especially temperature, salinity and pressure, might have affected the bacterial diversity.

In turn, the archaeal community composition (ACC) present in the eight selected samples was analysed by targeting the V1–V3 region of the archaeal 16S rRNA gene. Even the mentioned limitation on coverage of the selected primer combination for *Archaea* (see M&M section), archaeal sequences were obtained for all the production waters samples except the non-flooded production water PNFT1 sample. Interestingly, this sample had sequences exclusively related to *Pirellula*, a member of the *Planctomycetes* bacterial group that has been described to contain several archaeal-like genes [55].

Concerning the injection water (IS2, IBD and IT3), only bacterial sequences (mainly related to *Planctomycetes*, *Verrucomicrobia*, *OD1* classes and unclassified sequences) were retrieved with archaeal primers. Different reasons could justify the flaws of the archaeal analysis presented here: the presence of some specific archaeal groups not properly covered by the primers combination used, the low relative abundance of the *Archaea* in comparison with their bacterial counterpart. Another possibility is that our chemical procedure to disrupt the cells during nucleic acids extraction failed for some specific archaeal groups, mainly those present in injection water samples. However, the methods used successfully break the archaeal cells in the other samples collected in this study and has also been successfully applied in several and distinct water samples containing different proportions of *Archaea* and *Bacteria* [36], [56].

CARD-FISH analysis in injection waters samples (i.e., IS2, IBD and IT3), revealed that *Archaea* accounted for 7–30% of all hybridized cells. It’s not worth to mention that due to the application of different techniques, targeting distinct 16S rRNA gene regions and putative bias of the applied techniques, direct correlation between obtained results could not be performed. Furthermore, it has been recently evidenced that coverage values of general CARD-FISH probes were between 90% and 94% for *Archaea* and *Bacteria*, respectively [57]. However, and in order to reduce the flaws depicted above, the development of new primer combinations and probes with higher coverage are encouraging to shed some light into the *Archaea* domain in these kind of samples.

Archaeal sequences were analyzed using the same methods as for *Bacteria*. After denoising and quality checking, specifically, 1341 or 1754 sequences from the 3390 reads were classified as *Archaea* or *Bacteria*, respectively, whereas 295 sequences were unknown. The number of sequences per sample varied from 195 to 452 (Table S2B). We subsampled all the samples in Mothur randomly to the same size based on the sample with 195 sequences. Although observed species and CHAO1 estimations have changed numerically, our main conclusion with samples PFOH2, PFS1, PNFT2 and PFOH1 was same with the one produced with the original dataset. Therefore we presented here our original data. The rarefaction curve that reached the plateau level, indicating a good sequencing coverage, was obtained for the PFOH1 and PNFT2 samples (Fig. S1B). Furthermore, Shannon index indicated that the PFOH1 sample harbored the greatest diversity (Table S2B). These results provide insight into the archaeal diversity of these extreme ecosystems; nevertheless, very little is known about archaeal diversity in comparison to the bacterial diversity in these kind of environments until now. The problems apprehending archaeal diversity are due to the lack of cultured representatives for all archaeal groups and a dearth of full-length 16S rRNA gene sequences. With the development of sequencing technologies and the enlargement of the archaeal databases, better primers, probes and phylogenetic assignations should be available in the coming years.

### Analysis of Bacterial Communities

The relative abundance of bacterial OTUs at 97% was used to perform MDS and ANOSIM analysis, in order to highlight differences between water samples (injection versus production waters and production waters from flooded versus non-flooded wells). Procrustes analyses [58], [59] showed that bacterial diversity patterns were strongly reproducible at both taxonomic levels (correlation coefficient = 0.83, *p*<0.004).

ANOSIM analysis revealed a significant difference in bacterial community composition between injection and production waters (R = 0.477, *p*<0.05) as previously reported using DGGE, T-RFLP and 16S rRNA gene clone library analysis [4], [21], [23]. These results are not surprising as these samples have very different physicochemical properties. However, no significant differences were found between the BCC of production waters from flooded and non-flooded wells (R = 0, *p*>0.05), despite the differences observed in physicochemical characteristics and the fact that the samples are from different formations. This is consistent with the results obtained for cell abundance and bacterial and archaeal relative abundance.

**Figure 2:**
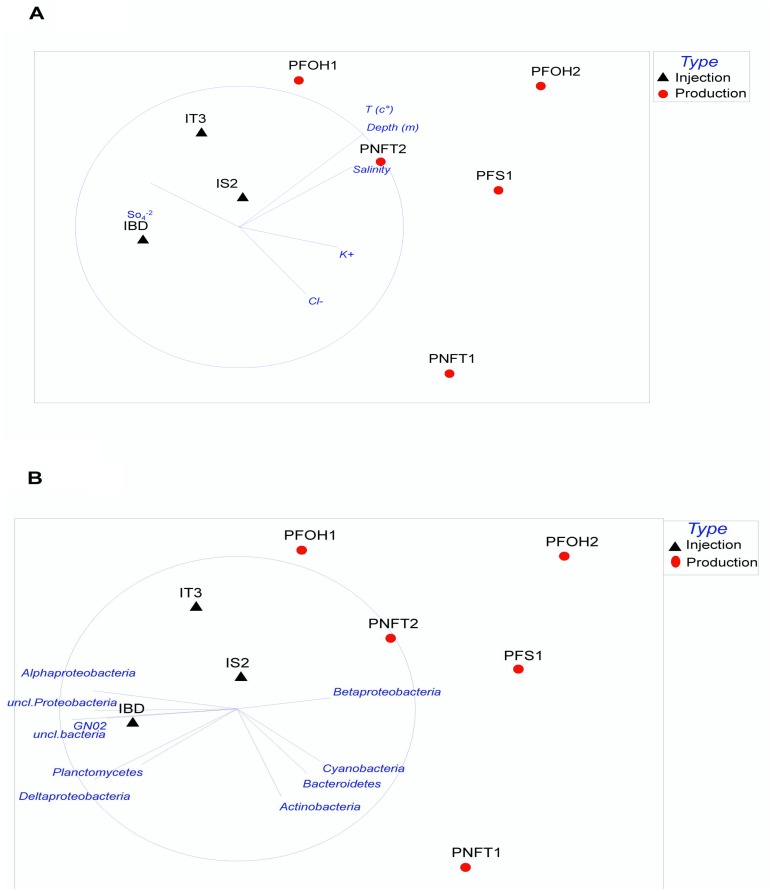
Nonmetric multidimensional scaling plot showing the relationship between the different environmental parameters (A)/phyla (B) and relative abundance of bacterial OTUs of studied water samples. Increasing distance between points equates to decreasing similarity in BCC. MDS plot are based on Bray Curtis distances generated from square root transformed data. Analysis was conducted using Primer 6.

The Spearman correlation coefficient was used to show the relationship between the different environmental parameters/phyla and relative abundance of bacterial OTUs. As illustrated in Figure 2A, BCCs in production waters (PNFT2, PFOH2, PFOH1, PFS1 and PNFT1) were significantly correlated with temperature, depth, salinity, Cl^−^ and K^+^, while BCCs in injection waters (IBD, IS2 and IT3) were significantly correlated with SO_4_^2−^. These results highlight the major impact of the physicochemical characteristics of production and injection waters on the BCC. Regarding the bacterial phylogenetic groups retrieved (Fig. 2B), the BCC of PNFT1 well was significantly correlated with *Actinobacteria*, *Cyanobacteria* and *Bacteroidetes*, while those of the PNFT2, PFOH2, PFOH1 and PFS1 wells were related to *Betaproteobacteria*. However, injection water samples (IBD, IS2 and IT3) showed a positive correlation with *Alpha*-, *Deltaproteobacteria* and unclassified *Proteobacteria.*

### Phylogenetic Assignation of the Bacterial Community

**Figure 3:**
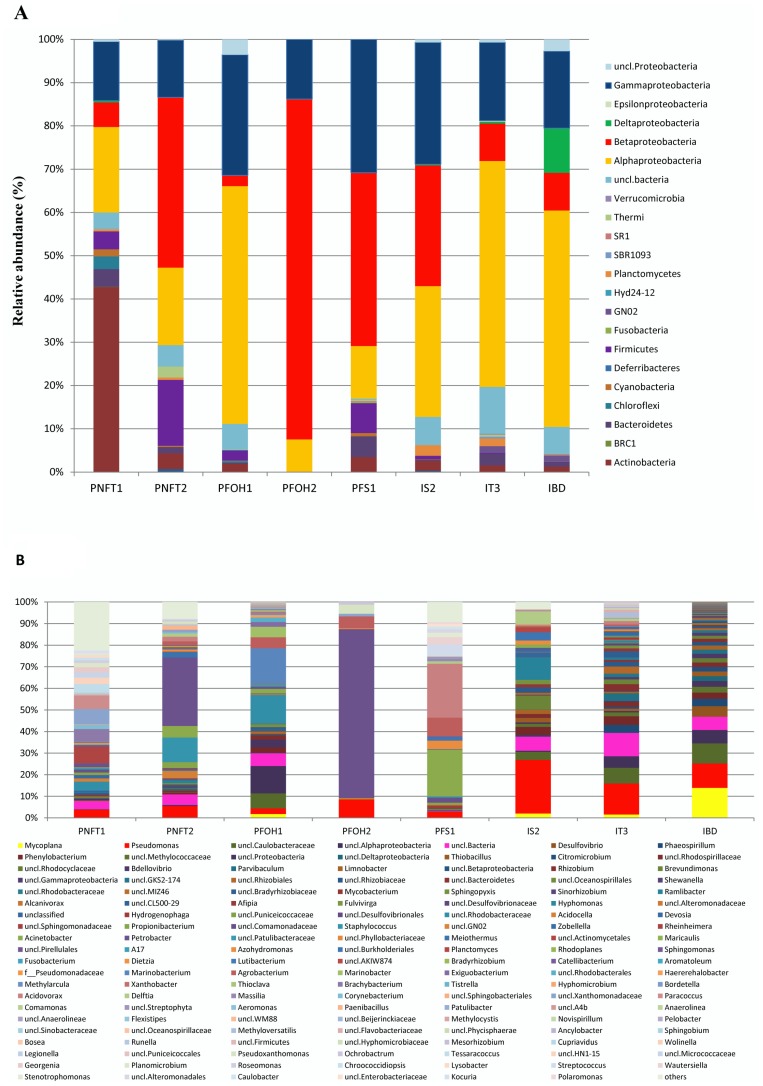
Taxonomic classification of bacterial reads retrieved from different wells at phylum (A) and genus (B) levels from 16S rRNA gene pyrosequencing.

With the exception of four sequences, all the qualified reads obtained using bacterial primers could be assigned to *Bacteria* using the greengenes database classifier at a confidence threshold of 80%. However, 38% of all the analyzed sequences were not assigned to any know bacterial group, suggesting that subsurface waters harbor a community of not yet described bacterial phyla. The relative abundance of specific bacterial groups was studied at different taxonomic levels, i.e., phylum, class, and genus. Altogether, 17 bacterial phyla were recovered from all the samples. The classification analysis of bacterial sequences is presented in Fig. 3A and Fig. 3B (See also Table S3A and Table S3B). *Proteobacteria* were found to be the most dominant phylum in all samples (except for PNFT1), ranging from 78 to 85% in injection waters and 69 to 98% in production waters. On average up to 8% of these sequences were unclassified *Proteobacteria*. The other phyla representing more than 1% of the sequences were *Actinobacteria*, *Firmicutes*, *Bacteroidetes*, *Chloroflexi*, *Thermi*, *Planctomycetes*, *Cyanobacteria*, *Acidobacteria*, *Candidate division GNO2*. The phyla *Defferibacteres*, *Fusobacteria, Verrucomicrobia* and uncultured candidate divisions (SBR1093, SR1, BRC1 and Hyd24-12) were also present but accounted for less than 1% of the sequences.

*Alpha-*, *Beta-* and *Gammaproteobacteria* predominated in seven samples. Sequences assigned to the *Alphaproteobacteria* class in injection waters accounted for 52%, 50% and 28% of total sequences in IT3, IBD and IS2, respectively. In production waters, this class was also very well represented with relative abundance of 56% in PFOH1, 12% in PFS1, 17% in PNFT2, 19% in PNFT1 and 7.5% in PFOH2. The most abundant genera related to *Alphaproteobacteria* in injection waters were: *Brevundimonas*, *Phaeospirillum*, *Sphingopyxis*, *Sinorhizobium*, *Rhizobium*, *Parvibaculum*, *Citromicrobium* and *Hyphomonas*. Some of these *Bacteria* have been described as hydrocarbon degraders [60], [61], [62]. Among the other *Alphaproteobacteria*, *Sphingomonas* and *Agrobacterium* were only found in production waters and were more abundant in PFS1. Some strains of *Sphingomonas* sp. are well known as PAH (polycyclic aromatic hydrocarbon) degraders [61], while Yingfei [63] reported that a *Agrobacterium tumefaciens* strain (UP3) has the ability to remove sulfur (biodesulfurization) from sulfur-containing petroleum. In the PNFT1 well, where the temperature was 87°C and pH 4.6, members of *Alphaproteobacteria* were different from those found in the other wells and assigned to the *Paracoccus*, *Devosia*, *Acidocella* and *Roseomonas* genera. Some *Paracoccus* strains are extremophile nitrate reducer and have already been found in Canadian oilfields [64] while an acidophilic strain of *Acidocella* sp. was isolated from acidic coal mine drainage [65] but not yet described in oilfields.

The relative abundance of *Betaproteobacteria* varied in production and injection waters without any particular trend. In the production waters, they accounted for 2.4% in PFOH1, 5% in PNFT1, 39% in PNFT2, 40% in PFS1 and 79% in PFOH2. In the injection waters, *Betaproteobacteria* accounted for 8.6%, 8.7% and 28% of total sequences in IT3, IBD and IS2, respectively. Among *Betaproteobacteria*, the genera *Thiobacillus* (nitrate-reducing and thiosulfate-oxidizing), *Limnobacter* (thiosulfate oxidizing), *Methyloversatilis* (methylotrophs), *Ramlibacter* and *Delftia* were found in the injection waters, while the genera *Acidovorax*, *Azohydromonas*, *Polaromonas* and *Hydrogenophaga* were mainly detected in PFS1 (production water). *Acidovorax* and *Hydrogenophaga* have already been detected in other oilfields [4], [5], [66], [67], whereas some species of *Thiobacillus, Polaromonas* have already been detected in different oil hydrocarbon-contaminated environmental samples [68]. *Bacteria* belonging to the *Petrobacter* genus were found in all the sampled production waters, but they were largely dominant in the PFOH2 (78%) and PNFT2 (31%) samples. The unique species of the genus *Petrobacter* described until now, *Petrobacter succinatimandens sp. nov*, was isolated from a non-water-flooded Australian terrestrial oil reservoir [69]. In our study, *Petrobacter* is a genus found in all the sampled production waters (water flooded and non-water-flooded wells), indicating that it is possibly indigenous to oil reservoirs.

*Gammaproteobacteria* accounted for 28%, 18% and 17.8% of total sequences in the IS2, IT3 and B-RN injection waters, respectively. In the production waters, *Gammaproteobacteria* were distributed as follows: PFS1 (30%), PFOH1 (27%), PFOH2 (13.8%), PNFT2 (13.6%) and PNFT1 (13.2%). Members of the *Gammaproteobacteria* that were detected in all samples were mainly *Pseudomonas*, *Acinetobacter*, *Marinobacterium*, *Marinobacter*, *Pseudoxanthomonas*, *Stenotrophomonas*, *Haererehalobacter* and *Shewanella*. *Pseudomonas* was present in seven samples, with higher relative abundance in injection waters, IS2 (24%), IT3 (14%) and IBD (11%). This genus has already been reported in oil reservoirs [4], [5], [66] and some strains of *Pseudomonas* are among the most widely studied oil degraders [70]. *Acinetobacter*, which was previously reported for bioremediation of oil spills, was profusely detected in PFS1 production water. *Marinobacter* and *Marinobacterium*, described as halophilic oil-utilizing *Bacteria* [60], were only present in PFOH1 production water, where the salinity was 5.8 g/L. It is interesting to note that these putative halophilic *Bacteria* were identified in wells having a salinity around six times lower salinity than the average salt content of seawater. *Shewanella* was detected only in IBD, one species of this genus (*S.putrefaciens)* has been reported as associated with oil pipelines and tank corrosion, formation of biofilms and has also been proposed as a candidate for bioremediation [71], [72].

*Deltaproteobacteria* were only highly abundant in the IBD well, which was characterized by a high sulfate concentration (2897 mg/L) and serious corrosion problems. It harbored *Desulfovibrio* and *Bdellovibrio* genera. Some species of *Desulfovibrio,* a sulphate-reducing *Bacteria*, has already been isolated from oilfields that had undergone biocorrosive damage [73], [74]. Since SRB are shown to be involved in MIC [7], presence of *Desulfovibrio* in the IBD well might have induced the corrosion problem observed in this well (Gana ML, personal communication). In addition to the important role they play in the sulphur and carbon cycle, some sulphate-reducing *Deltaproteobacteria* are capable of anaerobic degradation of benzene, naphthalene and other aromatic hydrocarbons with sulphate as the electron acceptor [75], [76].

The phylum Actinobacteria only predominated in PNFT1 (42%). Actinobacteria OTUs detected in PNFT1 were closely related to Arthrobacter, Brachybacterium, Tessaracoccus, Georgenia, Corynebacterium, Kocuria, Microbacterium, Propionobacterium and Dietzia. Most of these Bacteria have been found in other oilfields across the world and some are known to play an important role in biodegradation of various petroleum hydrocarbons [66], [70], [77], [78].

Among *Firmicutes,* the different genera were found to be specific to each well: *Streptococcus* in PFS1, *Exiguobacterium* in PFOH1, *Planomicrobium* in PNFT1 and *Paenibacillus* and *Staphylococcus* in PNFT2.

The phyla *Bacteroidetes* and *Planctomycetes* were respectively represented by *Wautersiella* (3.4%) and *Planctomycetes* (2.5%) in the IS2 well. Furthermore, the *Deinococcus/Thermi* phylum was mainly detected in PNFT2 including *Meiothermus*, a genus already isolated from geothermal areas [79].

*Cyanobacteria*, which accounted for 1% of the total bacterial sequences, was only detected in PNFT1 (temperature: 87°C, pH: 4.60 and salinity: 250 g/L) and was related to the *Chroococcidiopsis* genus. This bacterial genus is known for its ability to survive in the most extreme arid habitats including high temperature and high salinity [80]. These *Cyanobacteria* can form biofilms at the stone–soil interface under pebbles of desert pavements, colonize microscopic fissures or structural cavities (cryptoendoliths) of rocks, or grow within halite deposits [81]. However, the presence of this phototrophic organism in formation waters deserves further research. One hypothesis could be the development of a biofilm in the wellhead where light is accessible.

### Shared Bacterial Species

**Figure 4:**
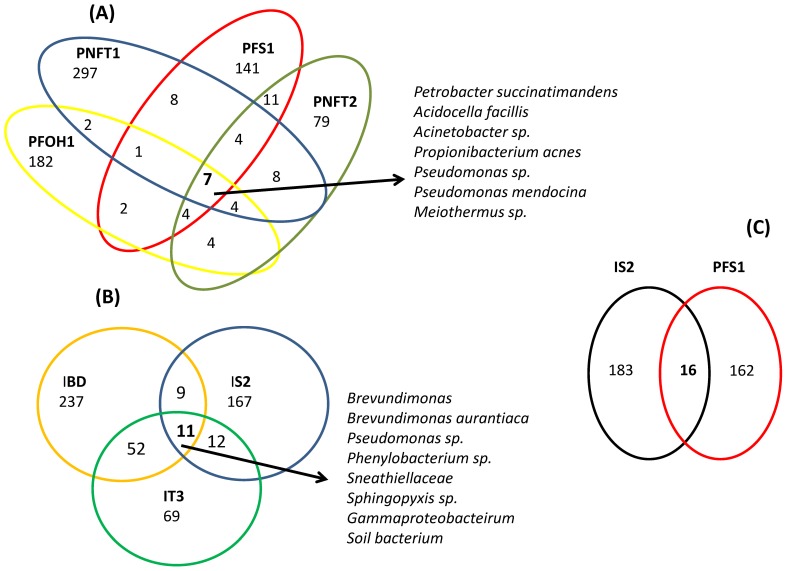
Venn diagram showing the shared bacterial OTUs (at a distance of 0.03) between all studied production waters (except PFOH2) (A), all studied injection waters (B) and production and injection waters from the same well (site S)(C).

Venn diagrams were constructed to evaluate the number and identity of the shared species between water samples from four production waters (PNFT1, PNFT2 PFOH1 and PFS1). PFOH2 was excluded because it is not diverse and only dominated by *Petrobacter* species. The diagram for the production waters indicates that the sum of total observed OTUs_0.03_ in the four samples was 836, but only seven OTUs_0.03_ were common to these wells (Fig. 4A). The low number of shared OTU confirmed previous studies, which reported that each production well had its own specific microbial composition [4]. *Petrobacter succintimandens*, *Acidocella facillis*, *Acinetobacter* spp., *Propionibacterium* spp., *Pseudomonas* spp., *Pseudomonas mendocina and Meiothermus* spp. were the species common to all the production waters studied, indicating that these species might be indigenous in the Algerian oil wells sampled. *Pseudomonas* and *Acinetobacter* relatives are commonly found in oil reservoirs [4], [82], [83], [84], even at high temperatures [5], [83]. Knowing that these species grow in mesophilic conditions in culture, Tang *et* *al*. [5] deduced that these species were mainly introduced by injection waters during oil production by long-term water flooding. In the present study, these two species were common to both high-temperature water-flooded and non-water-flooded petroleum reservoirs, suggesting that *Pseudomonas* and *Acinetobacter* might be indigenous species in oilfields and that they do not result from contamination by injection water. In addition, these wells are characterized by different salinity levels (ranging from 5.84 to 250 g/L) and pH (4.50 to 6.90), so isolating and culturing these specific genera might reveal new physiological and biochemical characteristics. However, complementary *in situ* sampling should be performed to be sure that these species have not grown in lower temperature regions of the wellhead infrastructure.

Regarding the subsurface injection water samples (IBD, IS2 and IT3), Venn diagrams (Fig. 4B) showed that only 11 out of 652 OTUs were common to these wells. These common OTUs were mainly affiliated to *Brevundimonas* spp., *Phenylobacterium* spp., *Pseudomonas* spp., *Sneathiellales* spp. and *Sphingopyxis* spp., which have been reported as related to oil degradation. Moreover, the distribution of sequences demonstrated once again that each subsurface water well has its own microbial population.

### Water Flooding Effect on Bacterial Community Composition in Petroleum Reservoir

The results presented here suggest that the *Bacteria* from injection waters do not significantly modify the BCC of the waters from flooded wells, probably because of strong selective pressure related to increasing depths.

To go one step further with this hypothesis, the BCC of the PFS1 production well was compared with the BCC of its injection water provided from the IS2 well. The Venn diagram for bacterial OTUs_0.03_ showed that only 16 OTUs out of 377 (4%) were common to both wells (Fig. 4C). Our results are consistent with the study conducted in oil wells in China, which indicated a shift in the BCC of injection waters after being introduced to the oil reservoir [4]. Even if the origin of the injection water differs (subsurface water in this study and surface waters in the study reported by Ren *et* *al.* [4], the results of both studies suggest that after the water was injected, only a few bacterial species could adapt to the extreme conditions of the petroleum reservoir.

### Diversity Analyses of the Archaeal Community

Archaeal sequences were only successfully amplified from four samples. Sequences of archaeal 16S rRNA genes from three of these samples (PFOH2, PNFT2, PFOH1) belonged to *Euryarchaeota* and were clustered into the classes, *Methanobacteria*, *Methanomicrobia* and *Halobacteria*, while in the PFS1 sample, all sequences were assigned to the *Crenarchaeota* phylum and the *Thermoprotei* class. The *Methanobacteria* and *Methanomicrobia* classes have already been reported in several other oilfields [1], [4], [5], [22], [84], [85], [86]; however, only a few studies have reported *Halobacteria* in petroleum reservoirs [87].

Furthermore, potential methanogens were also detected in the three water-flooded reservoirs samples (PFOH1, PFOH2 and PFS1), which contained high concentrations of sulphate ranging from 371 mg/L to 503 mg/L. Methanogenesis in presence of sulphate has already been reported in other environments [88]. In fact, Mitterer [88] suggested that the co-generation of hydrogen sulfide and methane could be related to the non-competitive usage of substrates to produce methane within the sulphate-reducing zone by methanogens.

**Figure 5:**
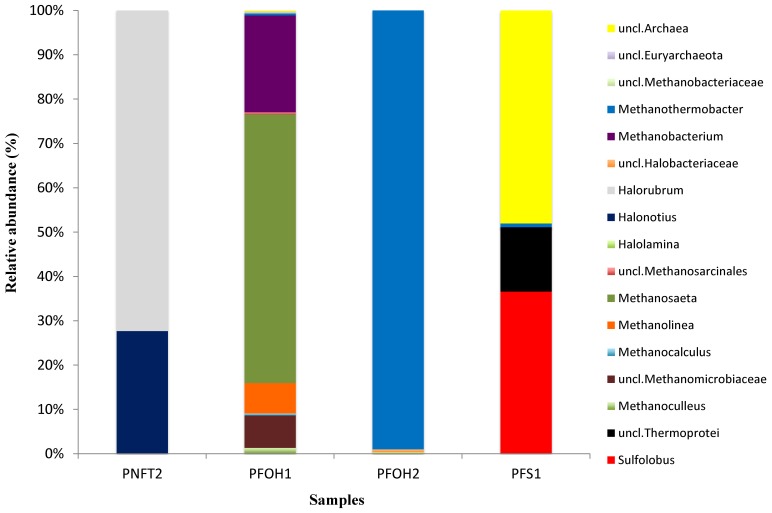
Taxonomic classification of archaeal reads retrieved from different wells at genus levels from 16S rRNA gene pyrosequencing.

Figure 5 (See also Table S4) showed the relative abundance of archaeal genera obtained in the present work. Clear differences in terms of relative abundance and phylogenetic groups were observed at the different sampling sites. *Methanothermobacter* and *Methanobacterium*, which are hydrogenotrophic methanogens, were found in PFOH2 and PFOH1 samples and account for 99% and 21% of the total archaeal sequences, respectively. In this sense, hydrogen-utilizing methanogens have been commonly recovered in high-temperature oil reservoirs, as in this study [1], [4], [5], [22], [89], [90].

In PFOH1, acetoclastic methanogens (*Methanosaeta*) and CO_2_-reducing methanogens (*Methanobacterium*, *Methanolinea*, *Methanoculleus* and *Methanocalculus*) coexist but with a large dominance of the obligate acetate-utilizer population. In fact, *Methanosaeta* accounted for 60% of the total archaeal sequences, whereas *Methanobacterium*, *Methanolinea*, *Methanoculleus* and *Methanocalculus* accounted for 21%, 7%, 1.3% and 0.5%, respectively. These genera had already been described in moderate- and high-temperature blocks in China [5]. Wang *et* *al*. [91] also reported that the metabolic types of the active methanogens varied substantially between reservoirs and appeared to be controlled by local physicochemical conditions within the reservoirs. In contrast to our study, previous studies showed that CO_2_-reducing methanogens identified from oilfield waters were more abundant than acetoclastic methanogens [20], [82], [92], [93]. In others studies based on experimental measurements of methanogenic pathways in oilfield waters, CO_2_ reduction to methane was more important than acetoclastic methanogenesis in petroleum reservoirs [94], [95]. Our results also contrast with the study of Warren *et* *al*. [96] suggesting the inhibition of acetolastic methanogenesis by the crude oil which would affect the overall methanogenic degradation rates of the petroleum hydrocarbons. However, one should keep in mind that the active community was not studied here, since the response of the total community to biotic and abiotic factors depends in part on whether individual phylotypes making up the community are active or not.

*Halorubrum* (72%) and *Halonotius* (28%), extremely halophilic *Archaea* relatives, were only abundant in PNFT2. In this sample, salinity reaches 190 g/L, which is five times greater than that of seawater. The *Halorubrum* genus in association with petroleum hydrocarbons was only previously described once in Russian samples [87], while *Halonotius* has never been described in oilfields. In fact, salt lakes and saltern crystallizer ponds were recently proposed as the designated habitats of the new *Halonotius* genus [97]. In our study, *Halonotius* was detected in water from 2104 m deep with pH 4.50, salinity 190 and a temperature 89°C. Cultivation is required to obtain more information on this genus inhabiting oilfields. An extreme halophilic *Archaea* has already been reported as a hydrocarbon degrader in an uncontaminated hypersaline pond [98] and in a contaminated saline-alkaline soil [99].

The thermophilic and hyperthermophilic *Archaea* such as *Sulfolobus* (36%) and *Thermoprotei* (14.5%) relatives were only detected in PFS1; 48% of the sequences in this sample were unclassified *Archaea*. *Sulfolobales* are usually found in geothermal areas where elemental sulphur is abundant, especially in volcanic hot springs where waters can be mildly (pH 4–6) or strongly acidic (pH 0.5–3), with temperature in the 60s to nearly 100°C and rich in sulphate [51]. *Sulfolobus*, which has never been reported in other oilfields, was the dominant genus in the PFS1 sample (T = 93°C, pH 6.08 and 371 mg/L of sulphate). *Thermoprotei* relatives were already described in production water from a high-temperature petroleum reservoir of an offshore oilfield in China [1]. Tang *et* *al*. [5] reported that species classified as *Thermoprotei* were only detected in production waters but not in injection waters, suggesting they are possibly indigenous to the oil reservoir. However, their metabolic properties are still unclear.

Compared to other studies, which reported that the dominant *Archaea* in all the oilfields studied were the methanogens and were probably the indigenous members of the oilfields [4], [5], [14], in the present work no methanogen was detected in saline and/or acidic wells, suggesting that archaeal community composition is determined by the physicochemical characteristics of the production waters of oil reservoirs.

### Conclusions

This study describes the composition and structure of bacterial and archaeal communities in different production and injection water samples from distant Algerian oilfields and wells. Tag-encoded FLX amplicon pyrosequencing (TEFAP) analyses were used to achieve deeper insight into their diversity.

Each well appeared to have its specific microbial population, driven by the physicochemical and geothermal conditions. A small number of shared taxa were nevertheless identified. Comparison of microbial composition in injection and production waters showed that they harbor a different microflora and that the *Bacteria* associated with the waters injected into the reservoir were not retrieved in the production waters, probably due to the extreme conditions encountered in those ecosystems. To the best of our knowledge, this is the first comparison of microbial community structure between production waters of flooded and non-water-flooded wells.

We found bacterial and archaeal genera that have already been reported in other oilfields across the world but also genera that had never been described in these ecosystems before. Many of the organisms identified are considered to be aerobic organisms suggesting limited knowledge about their full metabolic capabilities, further research about their development in anaerobic environments is thus necessary. For example, *Pseudomonas* was present in production waters from both flooded and non-water-flooded reservoirs, suggesting that this genus, considered until now as mainly introduced from injection water, might be indigenous to this ecosystem.

Many of the genera retrieved had already been reported as degraders of complex organic molecules and pollutants, but the metabolic potential of these communities is far from being completely understood and exploited. Cultivation efforts and functional metagenomic screening would be necessary to understand the metabolism of these communities and to exploit these specific and rich microflora for the purposes of biotechnology processes, notably for microbial enhanced oil recovery (MEOR).

## Supporting Information

Figure S1 **Rarefaction curves of bacterial (A) and archaeal (B) 16S rRNA genes, calculated by Mothur, indicating the numbers of operational taxonomic units (OTUs_0.03_) observed in the studied water samples.**
(TIFF)

Table S1 **Microbial abundance assessed by DAPI and CARD-FISH.**
(TIFF)

Table S2 **Estimation of OTU richness and diversity for the water samples studied. (A)** ***Bacteria*** **and (B)** ***Archaea*** **.** These indexes were calculated by Mothur.
(TIFF)

Table S3 **Relative abundance of bacterial phyla/class (A) and genera (B) expressed in percentages of the total number of bacterial sequences obtained by 16S rRNA gene pyrosequencing.**
(PDF)

Table S4 **Relative abundance of archaeal genera expressed in percentages of the total number of archaeal sequences obtained by 16S rRNA gene amplicon pyrosequencing**.
(TIFF)
