# Supplementary figures and images for: Diversity of Microbial Communities in Production and Injection Waters of Algerian Oilfields Revealed by 16S rRNA Gene Amplicon 454 Pyrosequencing
Source: PLoS One. 2013 Jun 21;8(6):e66588. doi: 10.1371/journal.pone.0066588 (PMC3689743; doi:10.1371/journal.pone.0066588)

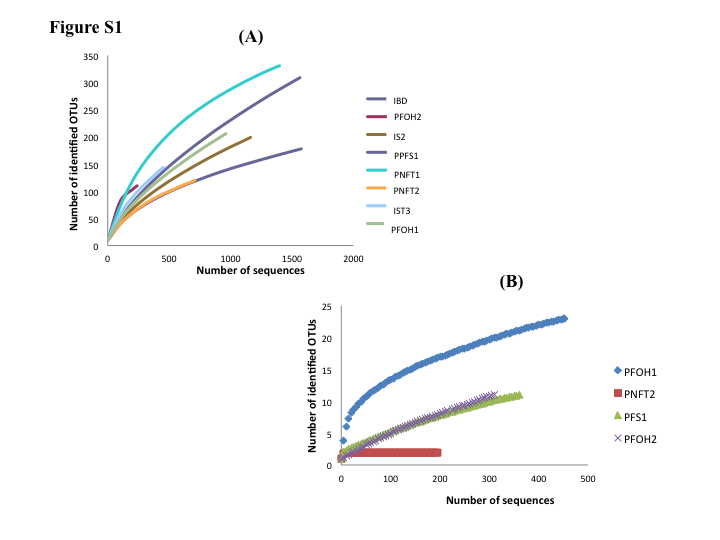

Supplement: Figure S1 — Rarefaction curves of bacterial (A) and archaeal (B) 16S rRNA genes, calculated by Mothur, indicating the numbers of operational taxonomic units (OTUs0.03) observed in the studied water samples. (TIFF) [file pone.0066588.s001.tiff]

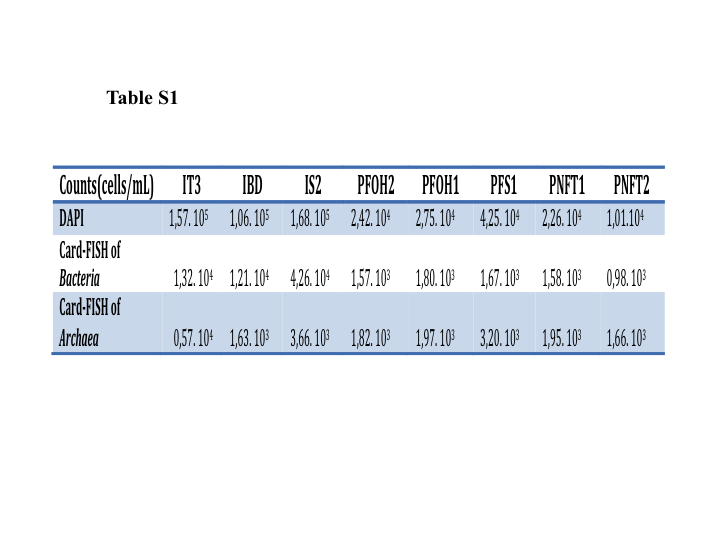

Supplement: Table S1 — Microbial abundance assessed by DAPI and CARD-FISH. (TIFF) [file pone.0066588.s002.tiff]

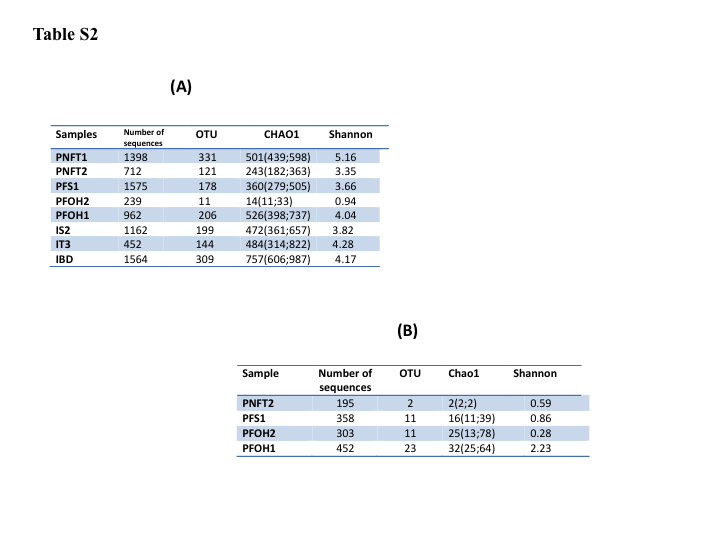

Supplement: Table S2 — Estimation of OTU richness and diversity for the water samples studied. (A) Bacteria and (B) Archaea . These indexes were calculated by Mothur. (TIFF) [file pone.0066588.s003.tiff]
