# Supplementary material for: Diversity of Microbial Communities in Production and Injection Waters of Algerian Oilfields Revealed by 16S rRNA Gene Amplicon 454 Pyrosequencing
Source: PLoS One. 2013 Jun 21;8(6):e66588. doi: 10.1371/journal.pone.0066588 (PMC3689743; doi:10.1371/journal.pone.0066588)
Supplement: Table S3 — Relative abundance of bacterial phyla/class (A) and genera (B) expressed in percentages of the total number of bacterial sequences obtained by 16S rRNA gene pyrosequencing. (PDF) [file pone.0066588.s004.pdf]

Table S3

(A)

**Phylum/class**	**PNFT1**	**PNFT2**	**PFOH1**	**PFOH2**	**PFS1**	**IS2**	**IT3**	**IBD**
**Acidobacteria**	0,00	0,71	0,00	0,00	0,00	0,34	0,00	0,00
**Actinobacteria**	42,78	3,67	1,98	0,00	3,49	2,24	1,55	1,28
**BRC1**	0,07	0,00	0,00	0,00	0,00	0,00	0,00	0,00
**Bacteroidetes**	4,08	1,41	0,21	0,00	4,76	0,34	2,65	1,02
**Chloroflexi**	2,93	0,00	0,31	0,00	0,00	0,00	0,00	0,00
**Cyanobacteria**	1,65	0,28	0,10	0,00	0,76	0,09	0,00	0,00
**Deferribacteres**	0,00	0,00	0,10	0,00	0,00	0,00	0,00	0,00
**Firmicutes**	4,15	15,23	2,29	0,00	6,92	0,78	0,22	0,13
**Fusobacteria**	0,00	0,00	0,00	0,00	0,25	0,00	0,00	0,06
**GN02**	0,00	0,00	0,00	0,00	0,00	0,00	1,55	1,34
**Hyd24-12**	0,00	0,00	0,10	0,00	0,00	0,00	0,00	0,00
**Planctomycetes**	0,43	0,56	0,00	0,00	0,00	2,41	1,77	0,19
**SBR1093**	0,00	0,00	0,00	0,00	0,00	0,00	0,44	0,19
**SR1**	0,00	0,00	0,00	0,00	0,19	0,00	0,00	0,00
**Thermi**	0,07	2,54	0,10	0,00	0,25	0,00	0,44	0,06
**Verrucomicrobia**	0,07	0,00	0,00	0,00	0,00	0,00	0,22	0,00
**uncl.bacteria**	3,79	4,94	5,93	0,00	0,32	6,55	10,84	6,14
**Alphaproteobacteria**	19,74	17,91	54,99	7,53	12,19	30,23	52,21	50,06
**Betaproteobacteria**	5,65	39,35	2,39	78,66	40,06	27,91	8,63	8,70
**Deltaproteobacteria**	0,43	0,00	0,10	0,00	0,00	0,26	0,44	10,29
**Epsilonproteobacteria**	0,00	0,00	0,00	0,00	0,00	0,00	0,22	0,00
**Gammaproteobacteria**	13,66	13,26	27,86	13,81	30,79	28,17	18,14	17,84
**uncl.Proteobacteria**	0,50	0,14	3,53	0,00	0,00	0,69	0,66	2,69

(B)

Genera	PNFT1	PNFT2	PFOH1	PFOH2	PFS1	IS2	IT3	IBD
**Mycoplana**	0,00	0,00	1,77	0,00	0,00	1,98	1,55	13,87
**Pseudomonas**	3,72	5,50	2,60	8,37	2,79	24,89	14,38	11,32
**uncl.Caulobacteraceae**	0,14	0,00	6,96	0,00	0,00	3,70	7,30	9,34
**uncl.Alphaproteobacteria**	0,14	0,42	12,68	0,00	0,06	0,52	5,31	6,20
**uncl.Bacteria**	3,79	4,94	5,93	0,00	0,32	6,55	10,84	6,14
**Desulfovibrio**	0,29	0,00	0,00	0,00	0,00	0,00	0,00	4,92
**Phaeospirillum**	0,00	0,00	0,21	0,00	0,00	0,69	3,76	3,45
**Phenylobacterium**	0,00	0,71	2,60	0,00	0,06	3,88	3,98	2,75
**uncl.Methylococcaceae**	0,07	0,00	0,00	0,00	0,00	1,38	1,77	2,75
**uncl.Proteobacteria**	0,50	0,14	3,53	0,00	0,00	0,69	0,66	2,69
**uncl.Deltaproteobacteria**	0,00	0,00	0,00	0,00	0,00	0,00	0,00	2,24
**Thiobacillus**	0,00	0,14	0,00	0,00	0,00	2,07	1,11	2,17
**Citromicrobium**	0,00	0,00	0,00	0,00	0,00	0,00	0,88	2,11
**uncl.Rhodospirillaceae**	0,00	0,85	1,77	0,00	0,00	1,81	2,21	2,05
**uncl.Rhodocyclaceae**	0,00	0,00	0,00	0,00	0,00	0,00	0,00	2,05
**Bdellovibrio**	0,14	0,00	0,00	0,00	0,00	0,00	0,44	1,98
**Parvibaculum**	0,00	0,42	0,00	0,00	0,00	0,00	3,54	1,92
**Limnobacter**	0,00	0,00	0,00	0,00	0,00	1,98	0,66	1,92
**uncl.Betaproteobacteria**	0,29	0,28	0,42	0,00	0,25	0,09	0,22	1,66
**Rhizobium**	0,00	0,00	0,31	0,00	0,00	0,00	3,32	1,47
**Brevundimonas**	0,72	0,28	0,10	0,00	0,63	6,55	2,21	1,41
**uncl.Gammaproteobacteria**	0,00	0,14	0,10	0,00	0,06	0,34	1,11	1,34
**uncl.GKS2-174**	0,00	0,00	0,00	0,00	0,00	0,00	1,55	1,21
**uncl.Rhizobiales**	0,21	0,00	1,04	0,42	0,06	0,86	3,32	0,90
**uncl.Rhizobiaceae**	0,00	0,71	0,52	0,00	0,13	2,41	2,21	0,90
**uncl.Bacteroidetes**	0,72	0,56	0,10	0,00	0,13	0,00	0,88	0,77
**uncl.Oceanospirillales**	0,00	0,00	0,10	0,00	0,00	0,00	0,00	0,77
**Shewanella**	0,00	0,00	0,00	0,00	0,00	0,00	0,00	0,77
**uncl.Rhodobacteraceae**	0,00	0,14	0,00	0,00	0,00	0,00	1,11	0,70
**uncl.MIZ46**	0,00	0,00	0,00	0,00	0,00	0,00	0,00	0,70
**uncl.Bradyrhizobiaceae**	0,93	0,28	1,25	0,00	0,00	0,09	2,88	0,58
**Mycobacterium**	0,00	0,00	0,10	0,00	0,13	1,46	1,33	0,58
**Sphingopyxis**	0,00	0,56	1,25	0,00	0,00	1,98	1,33	0,38
**Sinorhizobium**	0,00	0,00	0,00	0,00	0,00	0,00	0,88	0,38
**Ramlibacter**	0,21	1,27	0,31	0,00	0,00	10,34	0,22	0,32
**Alcanivorax**	0,00	0,00	0,00	0,00	0,00	0,00	0,22	0,32
**uncl.CL500-29**	0,00	0,00	0,00	0,00	0,00	0,00	0,00	0,32
**Afipia**	0,00	0,00	0,00	0,00	0,00	0,00	0,00	0,32
**Fulvivirga**	0,00	0,00	0,00	0,00	0,00	0,09	0,00	0,26
**uncl.Desulfovibrionaceae**	0,00	0,00	0,00	0,00	0,00	0,00	0,00	0,26

**Hyphomonas**	0,00	0,00	0,10	0,00	0,00	0,00	1,11	0,19
**uncl.Alteromonadaceae**	0,00	0,00	0,10	0,00	0,00	0,00	0,22	0,19
**unclassified**	0,72	0,56	0,10	0,00	0,00	2,41	0,00	0,19
**Hydrogenophaga**	0,00	0,42	0,00	0,00	1,14	0,00	1,11	0,19
**uncl.Puniceicoccaceae**	0,00	0,00	0,00	0,00	0,00	0,00	0,44	0,19
**uncl.Desulfovibrionales**	0,00	0,00	0,00	0,00	0,00	0,00	0,00	0,19
**uncl.Rhodobacteraceae**	4,22	0,00	12,99	0,00	0,00	0,00	0,44	0,13
**Acidocella**	1,50	3,53	0,31	0,42	0,25	0,17	0,00	0,13
**Devosia**	1,50	0,71	0,21	0,00	0,00	1,29	1,55	0,13
**uncl.Sphingomonadaceae**	0,07	0,56	0,10	0,00	0,00	0,00	0,44	0,13
**Propionibacterium**	1,14	2,68	0,10	0,00	1,02	0,00	0,00	0,13
**uncl.Comamonadaceae**	1,36	0,00	0,00	0,00	2,35	0,52	0,00	0,13
**Staphylococcus**	0,64	11,42	0,00	0,00	0,57	0,09	0,00	0,13
**uncl.GN02**	0,00	0,00	0,00	0,00	0,00	0,00	0,00	0,13
**Zobellella**	0,00	0,00	0,00	0,00	0,00	0,00	0,00	0,13
**Rheinheimera**	0,00	0,00	0,00	0,00	0,00	0,00	0,00	0,13
**Acinetobacter**	0,43	5,36	2,08	0,00	21,59	1,46	0,22	0,06
**Petrobacter**	1,72	31,73	1,04	78,24	0,06	0,00	0,00	0,06
**uncl.Patulibacteraceae**	0,00	0,00	0,94	0,00	0,00	0,00	0,00	0,06
**uncl.Phyllobacteriaceae**	0,00	0,00	0,10	0,00	0,00	0,09	0,66	0,06
**Meiothermus**	0,07	2,54	0,10	0,00	0,25	0,00	0,44	0,06
**uncl.Actinomycetales**	7,73	0,14	0,10	0,00	0,00	0,00	0,00	0,06
**Maricaulis**	0,00	0,00	0,10	0,00	0,00	0,00	0,00	0,06
**uncl.Pirellulales**	0,43	0,00	0,00	0,00	0,00	0,00	0,66	0,06
**A17**	0,00	0,00	0,00	0,00	0,00	0,00	0,44	0,06
**Azohydromonas**	0,00	0,99	0,00	0,42	3,94	1,81	0,00	0,06
**uncl.Burkholderiales**	0,50	0,56	0,00	0,00	1,65	3,88	0,00	0,06
**Planctomyces**	0,00	0,56	0,00	0,00	0,00	2,33	0,00	0,06
**Rhodoplanes**	0,00	0,56	0,00	0,00	0,00	0,17	0,00	0,06
**Sphingomonas**	0,00	0,28	0,00	0,00	0,32	0,00	0,00	0,06
**Fusobacterium**	0,00	0,00	0,00	0,00	0,06	0,00	0,00	0,06
**Dietzia**	0,93	0,00	0,00	0,00	0,00	0,00	0,00	0,06
**Lutibacterium**	0,36	0,00	0,00	0,00	0,00	0,00	0,00	0,06
**uncl.AKIW874**	0,00	0,00	0,00	0,00	0,00	0,00	0,00	0,06
**Bradyrhizobium**	0,00	0,00	0,00	0,00	0,00	0,00	0,00	0,06
**Catellibacterium**	0,00	0,00	0,00	0,00	0,00	0,00	0,00	0,06
**Aromatoleum**	0,00	0,00	0,00	0,00	0,00	0,00	0,00	0,06
**f__Pseudomonadaceae**	0,00	0,00	0,00	0,00	0,00	0,00	0,00	0,06
**Marinobacterium**	0,00	0,00	16,42	0,00	0,00	0,00	0,00	0,00
**Agrobacterium**	0,00	1,83	5,09	5,44	8,63	0,52	0,66	0,00
**Marinobacter**	0,00	0,00	4,89	0,00	0,00	0,00	0,00	0,00
**Exiguobacterium**	0,00	0,00	2,18	0,00	0,00	0,00	0,00	0,00
**uncl.Rhodobacterales**	0,07	0,00	2,08	0,00	0,00	0,00	0,00	0,00
**Haererehalobacter**	0,00	0,00	1,14	0,00	0,00	0,00	0,00	0,00

**Methylarcula**	0,00	0,00	0,83	0,00	0,00	0,00	0,00	0,00
**Xanthobacter**	0,00	0,00	0,73	0,00	0,00	0,00	0,00	0,00
**Thioclava**	0,00	0,00	0,62	0,00	0,00	0,00	0,00	0,00
**Brachybacterium**	5,94	0,00	0,52	0,00	0,00	0,00	0,00	0,00
**Tistrella**	0,00	0,00	0,42	0,00	0,00	0,00	0,00	0,00
**Hyphomicrobium**	0,00	0,00	0,31	0,00	0,00	0,09	0,00	0,00
**Bordetella**	0,00	0,00	0,21	0,00	0,00	0,00	0,00	0,00
**Acidovorax**	0,00	2,12	0,10	0,00	24,95	0,43	1,55	0,00
**Delftia**	0,00	1,55	0,10	0,00	1,14	6,12	1,11	0,00
**Massilia**	0,00	0,00	0,10	0,00	1,14	0,26	0,22	0,00
**Corynebacterium**	1,93	0,56	0,10	0,00	0,13	0,00	0,22	0,00
**uncl.Sphingobacteriales**	0,21	0,00	0,10	0,00	0,00	0,00	0,22	0,00
**uncl.Xanthomonadaceae**	7,15	0,85	0,10	1,26	0,00	0,00	0,00	0,00
**Paracoccus**	6,22	0,00	0,10	0,00	0,00	0,43	0,00	0,00
**Comamonas**	0,00	0,00	0,10	0,00	0,00	0,09	0,00	0,00
**uncl.Streptophyta**	0,00	0,28	0,10	0,00	0,76	0,00	0,00	0,00
**Aeromonas**	0,07	0,00	0,10	0,00	0,06	0,00	0,00	0,00
**Paenibacillus**	0,00	1,97	0,10	0,00	0,00	0,00	0,00	0,00
**Patulibacter**	0,00	0,00	0,10	0,00	0,00	0,00	0,00	0,00
**uncl.A4b**	0,00	0,00	0,10	0,00	0,00	0,00	0,00	0,00
**Anaerolinea**	0,00	0,00	0,10	0,00	0,00	0,00	0,00	0,00
**uncl.Anaerolineae**	0,00	0,00	0,10	0,00	0,00	0,00	0,00	0,00
**Flexistipes**	0,00	0,00	0,10	0,00	0,00	0,00	0,00	0,00
**uncl.WM88**	0,00	0,00	0,10	0,00	0,00	0,00	0,00	0,00
**uncl.Beijerinckiaceae**	0,00	0,00	0,10	0,00	0,00	0,00	0,00	0,00
**Methylocystis**	0,00	0,00	0,10	0,00	0,00	0,00	0,00	0,00
**Novispirillum**	0,00	0,00	0,10	0,00	0,00	0,00	0,00	0,00
**Pelobacter**	0,00	0,00	0,10	0,00	0,00	0,00	0,00	0,00
**uncl.Sinobacteraceae**	0,00	0,00	0,10	0,00	0,00	0,00	0,00	0,00
**uncl.Oceanospirillaceae**	0,00	0,00	0,10	0,00	0,00	0,00	0,00	0,00
**Methyloversatilis**	0,00	0,00	0,00	0,00	0,06	0,00	2,21	0,00
**uncl.Flavobacteriaceae**	0,50	0,14	0,00	0,00	0,13	0,09	1,33	0,00
**uncl.Phycisphaerae**	0,00	0,00	0,00	0,00	0,00	0,00	0,66	0,00
**Ancylobacter**	0,00	0,00	0,00	0,00	0,00	0,00	0,66	0,00
**Sphingobium**	0,79	0,56	0,00	0,00	0,00	0,00	0,22	0,00
**Bosea**	0,14	0,14	0,00	0,00	0,00	0,00	0,22	0,00
**Runella**	0,00	0,00	0,00	0,00	0,00	0,00	0,22	0,00
**uncl.Firmicutes**	0,00	0,00	0,00	0,00	0,00	0,00	0,22	0,00
**uncl.Hyphomicrobiaceae**	0,00	0,00	0,00	0,00	0,00	0,00	0,22	0,00
**Mesorhizobium**	0,00	0,00	0,00	0,00	0,00	0,00	0,22	0,00
**Cupriavidus**	0,00	0,00	0,00	0,00	0,00	0,00	0,22	0,00
**Wolinella**	0,00	0,00	0,00	0,00	0,00	0,00	0,22	0,00
**Legionella**	0,00	0,00	0,00	0,00	0,00	0,00	0,22	0,00
**uncl.Puniceicoccales**	0,00	0,00	0,00	0,00	0,00	0,00	0,22	0,00

**Pseudoxanthomonas**	0,00	0,99	0,00	4,18	0,38	0,00	0,00	0,00
**Ochrobactrum**	0,00	0,42	0,00	1,26	0,00	0,00	0,00	0,00
**Tessaracoccus**	3,79	0,00	0,00	0,00	0,00	0,00	0,00	0,00
**uncl.HN1-15**	2,93	0,00	0,00	0,00	0,00	0,00	0,00	0,00
**uncl.Micrococcaceae**	2,43	0,14	0,00	0,00	0,00	0,00	0,00	0,00
**Georgenia**	2,43	0,00	0,00	0,00	0,00	0,00	0,00	0,00
**Planomicrobium**	1,93	0,00	0,00	0,00	0,00	0,00	0,00	0,00
**Roseomonas**	1,22	0,42	0,00	0,00	0,00	0,00	0,00	0,00
**Chroococcidiopsis**	1,22	0,00	0,00	0,00	0,00	0,00	0,00	0,00
**Lysobacter**	1,14	0,00	0,00	0,00	0,00	0,00	0,00	0,00
**Streptococcus**	0,00	0,14	0,00	0,00	5,08	0,00	0,00	0,00
**Wautersiella**	0,00	0,00	0,00	0,00	3,43	0,00	0,00	0,00
**Stenotrophomonas**	0,57	0,00	0,00	0,00	2,10	0,00	0,00	0,00
**uncl.Alteromonadales**	0,00	0,00	0,00	0,00	1,59	0,00	0,00	0,00
**Caulobacter**	0,00	0,00	0,00	0,00	1,27	0,00	0,00	0,00
**uncl.Enterobacteriaceae**	0,29	0,00	0,00	0,00	1,08	0,00	0,00	0,00
**Kocuria**	1,36	0,00	0,00	0,00	0,00	0,09	0,00	0,00
**Polaromonas**	0,00	0,00	0,00	0,00	0,95	0,00	0,00	0,00
**others**	22,46	7,90	0,00	0,00	9,27	3,36	0,00	0,00
